# Detecting Minimal Hepatic Encephalopathy in an Endemic Country for Hepatitis B: The Role of Psychometrics and Serum IL-6

**DOI:** 10.1371/journal.pone.0128437

**Published:** 2015-06-03

**Authors:** Chia-Fen Tsai, Chi-Jen Chu, Yi-Hsiang Huang, Yen-Po Wang, Pei-Yi Liu, Han-Chieh Lin, Fa-Yauh Lee, Ching-Liang Lu

**Affiliations:** 1 Institute of Brain Science, School of Medicine, National Yang-Ming University, Taipei, Taiwan; 2 Department of Medicine, School of Medicine, National Yang-Ming University, Taipei, Taiwan; 3 Division of Gastroenterology, Taipei Veterans General Hospital, Taipei, Taiwan; 4 Department of Psychiatry, Taipei Veterans General Hospital, Taipei, Taiwan; University of Navarra School of Medicine and Center for Applied Medical Research (CIMA), SPAIN

## Abstract

**Background & Aims:**

It remains unknown what the prevalence of minimal hepatic encephalopathy is in Taiwan, a highly endemic country for chronic viral hepatitis infection. It is also unclear whether abnormal serum cytokine levels can be indicative of the presence of minimal hepatic encephalopathy. We aimed to standardize the tests of psychometric hepatic encephalopathy score and predictive value of proinflammatory cytokines in minimal hepatic encephalopathy in Taiwan.

**Methods:**

180 healthy subjects and 94 cirrhotic patients without a history of overt hepatic encephalopathy from a tertiary center were invited to participate in this cross-sectional study. Blood sampling for determination of serum levels of interleukin 6 and 18 and tumor necrosis factor-α was performed. Based on the normogram of psychometric hepatic encephalopathy score from healthy volunteers, patients with minimal hepatic encephalopathy were identified from the cirrhotic patients using the criterion of a psychometric hepatic encephalopathy score less than −4.

**Results:**

In the healthy subjects, age and education were predictors of subtests of psychometric hepatic encephalopathy score. Minimal hepatic encephalopathy was identified in 27 (29%) cirrhotic patients. Serum interleukin 6 level (OR = 6.50, 95% CI = 1.64–25.76, *P* = 0.008) was predictive of the presence of minimal hepatic encephalopathy after multivariate analysis.

**Conclusions:**

The psychometric hepatic encephalopathy score can be a useful tool for detecting patients with minimal hepatic encephalopathy in Taiwan and around one third of cirrhotic outpatients fulfill this diagnosis. A high serum interleukin 6 level is predictive of the presence of minimal hepatic encephalopathy.

## Introduction

Hepatic encephalopathy (HE) comprises of a broad spectrum of neuropsychiatric abnormalities of varying severity, and affected patients usually suffer from psychomotor, cognitive, emotional, behavioral, and motor coordination dysfunctions [1]. Patients with minimal HE (MHE), a subclinical form of HE, usually have a normal mental and neurological status upon routine clinical examination. The subtle deficits in patients with MHE can only be elicited by specialized neuropsychological tests [2]. Despite the subclinical presentation of MHE, MHE patients are a significant burden on society and are burdened themselves, because they are more likely to suffer from car accidents, falls, a lower quality of life, and overt HE (OHE) as well as increased risk of requiring health care, hospitalization and even overall mortality [3–5]. The pathogenesis of MHE has not been fully clarified, but neuroinflammation may play a pivotal role. For example, serum interleukin 6 (IL-6) and IL-18 cytokines levels correlate with the grade of MHE [6]. Rodent studies also show that MHE rats display neuroinflammation and cognitive and motor alterations which can be reversed with anti-inflammatory drugs [7]. Despite this fact, it remains unknown whether abnormal serum cytokine levels can be predictive of the presence of MHE among cirrhotic patients.

Taiwan is highly endemic for chronic viral hepatitis infection, with viral hepatitis B seroprevalence of 17.3% in the general population. These factors led to chronic liver disease/cirrhosis being the ninth leading cause of death in Taiwan in 2012 [8]. Thus, early detection and subsequent treatment for MHE among cirrhotic patients in Taiwan is crucial to minimize the negative effect of MHE. Evidence have recommended using the Psychometric Hepatic Encephalopathy Score (PHES) as an efficient tool for detecting MHE [2,9]. The PHES combines 5 subtests: the digit symbol test (DST), number connection test-A (NCT-A), number connection test-B (NCT-B), serial dotting test (SDT), and line tracing test (LTT), to assess psychomotor speed and accuracy, visual perception, visuospatial orientation, visual construction, concentration, attention, and working memory [2]. Patients with MHE can be reliably distinguished from patients with OHE and from healthy controls by using this simple ‘paper and pencil’ test [2,10–15]. For patients awaiting liver transplantation, the PHES has also been demonstrated to be the most robust method for HE diagnosis and follow-up [16]. Because the results of the PHES can be affected by multiple factors, such as age, gender, and educational status, an age-corrected normogram may differ among different tested populations. The International Society on Hepatic Encephalopathies and Nitrogen Metabolism has suggested that MHE should be diagnosed using standardized tests and that results should be validated for the respective study population [17]. The PHES has been standardized in countries of low and intermediate endemicity for hepatitis B infection such as Germany [2], Italy [10], Spain [11], India [12], Mexico [13], South Korea [14], and China [15]. Furthermore, in previous studies with the application of PHES in other countries, many of their cirrhotic were alcoholism in etiology (49% in Italy, 42.1% in Spain, 49.4% in India, 21.1% in Korea, and 11% in Mexico) [10–14] Alcoholism *per se* might also affect cognitive function,[18] which would cause a bias in the recognition of MHE. In addition, previous studies were conducted in the countries with low or medium epidemic for hepatitis B. To date, PHES has not been applied in cirrhotic patients in highly endemic area for chronic hepatitis B, such as Taiwan. Thus, the aim of this study is to (1) standardize the PHES to a healthy population in Taiwan; (2) evaluate the prevalence of MHE among cirrhotic patients in Taiwan; (3) identify whether proinflammatory cytokines could be independently predictive factors for MHE occurrence.

## Materials and Methods

### Study Population

This study was conducted from January 2011 to March 2014 at outpatient clinic in a tertiary 2,700-bed referral center in Northern Taiwan. Two samples of subjects were invited to participate in the study. A sample of healthy adult Taiwanese (> = 20-year-old) were enrolled for the purpose of creating a standardized PHES. Another sample was composed of consecutive patients with liver cirrhosis to compare their PHESs to those of the Taiwan PHES standardizations. The study protocol was reviewed and approved by the institutional review board of Taipei Veterans General Hospital. (IRB No: 201101008OB) Written informed consent was obtained from the all participants or their legally authorized representatives according to the Declaration of Helsinki.

### PHES standardization sample

Advertisement posters were used to invite healthy subjects to participate in the study as standardized samples. Those who accompanied the cirrhotic patients to the clinics were also allowed to join. The following exclusion criteria were applied: abnormal results for liver or renal function; presence of chronic liver or renal, neurological or psychiatric diseases, or other diseases that can affect cognitive function; a history of chronic liver disease or a neurologic or psychiatric disorder; alcohol abuse (> 60 g/day for men and > 30 g/day for women); a history of psychotropic drug consumption.

### Cirrhotic patients sample

Patients with liver cirrhosis but without a history of OHE were enrolled [9]. Cirrhosis was diagnosed by liver biopsy or by the presence of biochemical, ultrasonographic, or endoscopic features of portal hypertension and/or liver dysfunction.The exclusion criteria included: presence of OHE; taking lactulose, antibiotics, or psychoactive drugs; presence of significant comorbidity including heart, respiratory, or renal failure; presence of neurological or psychiatric diseases; presence of hepatocellular carcinoma or other malignancy; alcohol abuse within the past 3 months (> 60 g/day for men and > 30 g/day for women). Blood samples of all the cirrhotic patients were taken for the etiological diagnosis of liver cirrhosis, including hepatitis B surface antigen (HBsAg) and antibody for hepatitis C virus (antiHCV). Serum samples were also saved for further testing, including antinuclear antibody, liver kidney microsomal antibody, and ceruloplasmin, if the etiology was not detected at these initial tests. History was also noted for any hepatotoxic drug intake, including homeopathic and herbal medications. An ultrasonography was also performed later on. Cryptogenic cirrhosis connoted the absence of serologic evidence of other causes of liver disease or a history of significant alcohol use. Blood sampling was also performed for liver function tests, hematologic parameters and serum proinflammatory cytokine measurements. Hepatic function of the cirrhotic patients was graded according to the Child–Pugh classification [19].

### Cytokine measurements

Blood sample (10 mL) was collected using BD Vacutainer tubes (RSTs; BD, Franklin Lakes, NJ) and was centrifuged at 1000 g for 15 minutes. The supernatants were collected and stored at -80°C until use. IL-6, IL-18, and tumor necrosis factor-α (TNF-α) levels were quantified using commercially available ELISA kits (Bender MedSystems, Vienna, Austria).

### Neuropsychological Testing

#### Psychometric Hepatic Encephalopathy Score

The 5 tests composing the PHES were provided to C.F. Tsai by Dr. Karin Weissenborn. The DST results were measured as points; those of the NCT-A, NCT-B, and SDT were measured as seconds. The line tracing test results were measured as both time to complete (LTTt, second) and as an error score (LTTe), as described previously [20,21], and LTT was defined as sum of LTTt and LTTe. The PHES procedure was repeated in a 30 sub-sample randomly selected from healthy volunteers at 4 weeks after the initial tests.

Variables affecting PHES results were included in multiple linear regression models to assess the magnitude of the effects, and variables with large standardized coefficients were included in subsequent multiple linear regression analyses. The unstandardized beta coefficients of these analyses were used in the final formulae to correct for these factors. These formulae were subsequently used to predict values for cirrhotic patients, and the difference between the predicted and observed results for each test was divided by the corresponding standard deviation (SD) of the reference population to obtain the deviation from “normal” as a multiple of the SD. Finally, differences for each test in multiples of the SD were summed as follows: a result≤1 SD above the predicted value was scored as + 1; results -1 SD and -2 SDs below the predicted value were scored as -1 and -2, respectively; a result ≤ -3 SDs was scored as -3. Thus, the sum of scores for the PHES ranged from +5 to -15. Cirrhotic patients exhibiting a PHES of < -4 SD were considered to have MHE.

#### Hospital Anxiety and Depression Scale (HADS)

Prior studies have shown that a significant proportion of patients with liver cirrhosis suffer from depression, which would affect their cognition [22]. Thus, we measured the psychological distress using the Taiwanese version of the HADS, which has been developed for non-psychiatric medical patients [23].

### Statistical analysis

Results are expressed as the mean ± SD. Bivariate analyses were performed using the Pearson correlation, Student’s *t*-test, Mann–Whitney U-test, and chi-square test, as appropriate. PHES results from healthy volunteers were used to compile reference values with which results for the cirrhotic population were compared. Using bivariate analyses of the effects of variables, factors that affected the results of each test in the PHES were identified. Test-retest and inter-rater reliability of the PHES were assessed using the intra-class correlation coefficient. Variables shown by univariate analysis to affect the outcome of the PHES total score and the diagnosis of MHE were included in multiple regression models. Age, years of education, IL-6, IL-18, and Child-Pugh scores were included in the multiple linear regression analysis with PHES as the dependent variable. A logistic regression analysis was conducted with the presence of MHE as the dependent variable and age, education level, IL-6, IL-18, and Child-Pugh category as independent variables. The cut-off level of IL-6 and IL-18 were determined from the healthy control group to be the mean plus three SDs. A two-tailed *P* value of <0.05 was considered statistically significant. Analyses were performed using SPSS version 15.0 software (SPSS Inc., Chicago, IL).

## Results

### PHES standardization sample

A total of 180 healthy subjects were enrolled in this study.(Table 1) The age distribution of participants was as follows: 20–29 years, 32 (17.78%); 30–39 years, 29 (16.11%); 40–49 years, 32 (17.78%); 50–59 years, 43 (23.89%); 60–69 years, 37 (20.56%), and 70–79 years, 7 (3.89%). Age and years of education showed positive (DCT-A/B, SDT, LTT) or negative (DST) correlations with all individual PHES tests, while gender was negatively associated with the NCT-A/B (Table 2). No association was found between the HADS score and any PHES test. The predictive equations were generated on the relating factors and used to compute the standard deviations and to calculate scores for each test (Table 3). The PHES in the standardization sample was -0.17±1.24 (median, 0; range: -5 to 3) and was not correlated with age (*P* =. 30), gender (*P* =. 38), education (*P* =. 54), or the HADS score (*P* =. 26).

**Table 1:** Clinical and demographic characteristics of controls and cirrhotic patients.

	Controls	Cirrhosis	*P*
	(N = 180)	(N = 94)	
**Age(years)**	46.58±15.04	58.98±9.34	<.001
**Gender(Male)**	87 (48.33%)	54 (57.45%)	0.16
**Education(years)**	14.03±2.78	10.21±3.70	<.001
**PHES: DST**	60.44±13.25	42.21±14.19	<.001
**PHES: NCT-A**	34.36±13.47	57.88±29.76	<.001
**PHES: NCT-B**	79.23±43.32	144.20±80.73	<.001
**PHES: SDT**	74.17±26.54	94.95±41.45	<.001
**PHES: LTT**	122.08±32.75	166.09±45.72	<.001
**PHES total score**	-0.17±1.24	-2.31±3.45	<.001
**IL-6 (pg/ml)**	1.48±0.20	2.42±1.25	<.001
**IL-18 (pg/ml)**	102.94±54.63	113.00±128.51	<.001
**TNF-α**	29.24±17.83	47.54±53.50	<.001
**HADS score**	9.22±5.85	9.68±6.90	0.59
**Etiology of liver cirrhosis:** B/C/dual B and C/alcohol/ cryptogenic	-	50/30/4/5/5	
**Child-Pugh A/B/C**	-	80/13/1	
**Child-Pugh score**	-	5.55±1.27	

Digit symbol test(DST); Number connection test-A (NCT-A); Number connection test-B (NCT-B); Serial dotting test (SDT); Line tracing test (LTT); psychometric hepatic encephalopathy score (PHES); Interleukin-6 (IL-6); Interleukin-18 (IL-18);Tumor necrosis factor-α (TNF-α); Hospital Anxiety and Depression Scale (HADS)

**Table 2:** Correlation coefficients between psychometric tests and age, gender, years of education, and HADS score in healthy subjects.

	DST	NCT-A	NCT-B	SDT	LTT
**Age(years)**	-0.65**	0.59**	0.66**	0.41**	0.49**
**Gender(male)**	-0.03	-0.17*	-0.15*	0.04	0.01
**Education(years)**	0.53**	-0.46**	-0.45**	-0.29**	-0.26**
**HADS**	0.01	-0.04	0.02	0.06	0.07

*P<0.05

**P<0.001

Digit symbol test(DST); Number connection test-A (NCT-A); Number connection test-B (NCT-B); Serial dotting test (SDT); Line tracing test (LTT); Hospital Anxiety and Depression Scale (HADS)

**Table 3:** Equations for predicting test results from gender, age and education years.

Test	Equation	SD
DST	53.84-(Age×0.43)+(Edu×1.91)	13.25
NCT-A	42.44+(Age×0.41)+(Edu×1.79)-(Gender×3.61)	13.47
NCT-B	87.92+(Age×1.42)-(Edu×4.78)-(Gender×14.70)	43.32
SDT	81.80+(Age×0.59)-(Edu×2.50)	26.54
LTT	125.08+(Age×0.86)-(Edu×3.01)	32.75

Gender: 0(female); 1(male); Digit symbol test(DST); Number connection test-A (NCT-A); Number connection test-B (NCT-B); Serial dotting test (SDT); Line tracing test (LTT); Standard deviation (SD)

### Cirrhotic patients sample

The clinical and demographic data of a total of 94 cirrhotic patients were showed in Table 1. The PHES in the cirrhotic group was correlated with age (r = -0.29, *P* =. 005), education-year (r = 0.32, *P* =. 002), Child–Pugh score (r = -0.34, *P* =. 001), serum IL-6 level (r = -0.40, *P* =. 005), and serum IL-18 (r = -0.41, *P* =. 005), but not with gender (*P* =. 94), HADS score (*P* =. 05) or serum TNF-α level (*P* =. 63). Multivariate linear analysis showed that IL-6 (**β** = -0.32, *P* =. 01) and years of education (**β** = 0.35, *P* =. 01) were associated with PHES score.

### Prevalence of MHE

MHE was detected in 27 (28.7%) out of the 94 cirrhotic patients. The proportion of patients with MHE progressively increased with the Child–Pugh grade (18 patients in grade A (22.50%); 8 in grade B (61.54%); and 1 in grade C (100.00%)). The correlation between IL-6 levels and the presence of MHE were found in both patients with HBV related cirrhosis(r = 0.54, *P* =. 003) and those with etiologies of other causes (r = 0.45, *P* =. 04). Cirrhotic patients with MHE had an older age, lower mean years of education, a higher serum IL-6 and IL-18 levels, and a higher Child–Pugh score than their non-MHE counterparts (Table 4). Multiple logistic analysis showed that years of education (OR = 0.21, 95% CI = 0.05–0.80) and IL-6 levels (OR = 6.50, 95% CI = 1.64–25.76) were independently predictive of a diagnosis of MHE as defined by PHES (Table 5).

**Table 4:** Clinical characteristics of cirrhotic patients with and without minimal hepatic encephalopathy (MHE).

	MHE(N = 27)	Non-MHE(N = 67)	*P*
**Age(years)**	63.44±10.46	57.18±8.27	0.008
**Gender(male)**	55.56%	58.21%	0.82
**Education(years)**	8.07±3.13	11.07±3.58	0.001
**Child-Pugh score**	6.15±1.38	5.32±1.15	0.009
**Child-Pugh A/B/C**	18/8/1	62/5/0	0.007
**IL-6**	3.17±1.63	1.98±0.69	0.008
**IL-18**	168.72±162.71	77.17±86.34	0.02
**TNF-α**	58.14±74.16	41.38±36.82	0.38
**HADS score**	10.37±4.93	9.39±7.58	0.47

Interleukin-6 (IL-6); Tumor necrosis factor-α (TNF-α); Hospital Anxiety and Depression Scale (HADS).

**Table 5:** Risk factors associated with the diagnosis of minimal hepatic encephalopathy as defined by the psychometric hepatic encephalopathy score.

	β	OR(95% CI)	*P*
**Interleukin-6**	1.87	6.50(1.64–25.76)	0.008
**Interleukin-18**	-	-	0.78
**Education**	-1.58	0.21(0.05–0.80)	0.02
**Age**	-	-	0.28
**Child-Pugh category**	-	-	0.17

Education level: 0(0–6), 1(7–12), 2(over 12 years)

Age: 0(20–29), 1(30–39), 2(40–49), 3(50–59), 4(60–69), 5 (over 70 years)

Child-Pugh: 0(A), 1(B), 2(C)

### Reliability and test-Retest

Regarding the repeatability of PHES, the results showed the correlations between the baseline and 4-week follow-up of the PHES tests were excellent. The intra-class correlation coefficients of the DST, NCT-A, NCT-B, SDT, and LTT between the baseline and 4-week follow-up data were, respectively: 0.93 (95% CI = 0.84–0.97, *P* <. 001), 0.91 (95% CI = 0.81–0.96, *P* <. 001), 0.88 (95% CI = 0.74–0.95, *P* <. 001), 0.73 (95% CI = 0.41–0.88, *P* =. 001), and 0.82 (95% CI = 0.60–0.92, *P* <. 001). Improved performance was noted after re-test in all 5 PHES tests. (Results of the 5 neuropsychological test/re-tests were: DST: 60.38±13.24 vs. 65.77±14.60 points; NCT-A: 34.37±13.45 vs. 26.33±11.41 s; NCT-B: 79.21± 43.34 vs. 63.43 ± 28.42; SDT: 74.24± 26.55 vs. 63.77±12.77 s; LTT: 122.13±32.87 vs. 114.73±41.81 points).

## Discussion

In the current study, we set up a standardization of the PHES in healthy Taiwanese individuals. Around one third of our cirrhotic outpatients had MHE, and the serum level of IL-6 was predictive of the presence of MHE in Taiwanese cirrhotic patients.

In the standardization sample, we found that all 5 subtests of the PHES were affected by age and years of education. Age and years of education are widely recognized to be associated with PHES performance [2,10–15]. Gender differences in PHES performance have been noted in validation studies from Spain, Mexico, and South Korea. In our study, we also demonstrated that male subjects showed better results in the NCT-A and NCT-B subtests [11]. The Mexican study [13] revealed a gender effect on the DST and SDT subtests, whereas the South Korean study [14] reported that the SDT subtest was more favorable for women than for men. However, no such gender difference was noted in any of the PHES tests in the studies from Italy [10] or China [15]. In addition, we found no association between the level of mood symptoms measured by the HADS and any of the 5 PHES tests. None of the factors including age, years of education, gender and HADS were found to be independently predictive of PHES after multiple linear regression analysis.

Weissenborn *et al*. were the first to show that the PHES exhibited optimal sensitivity and specificity for diagnosing MHE when the diagnostic cut-off was set at -4 [2]. This PHES criteria was employed by many subsequent studies of screening for MHE [2,10,11,13,14,20,24]. By using the same cut-off in the current study, we found that nearly one third of our cirrhotic patients (29%) were identified to meet the criteria for an MHE diagnosis. This prevalence among cirrhotic patients is in agreement with those in previous reports from Western countries (15–31%) [10,11,13]. When compared with the MHE prevalence from other Asian countries, our result was similar to the reports from South Korea (26%), [14] but lower than those from China (49%) [15] and India (48%) [12]. The severity of the chronic liver disease may result in this discrepancy, because the majority of cirrhotic patients in this study (85%) and the Korean study (81%) had a Child-Pugh grade A status, while only 45% and 22% of patients had this status in the Chinese and Indian studies, respectively. Previous studies shown that the MHE prevalence was increasing with Child-Pugh grade [11,15]. We also demonstrated that MHE patients have a higher trend for Child-Pugh score, though the case numbers in each Child-Pugh category were too small to make a firm conclusion. Nearly one-third of the present sample (patients with cirrhosis of mild severity) had MHE in Taiwan. We should be cautious in taking care of these cirrhotic patients without MHE since the severity of chronic liver disease is expected to progress. Some patients may eventually develop MHE.

The pathogenesis of HE is heterogeneous. Hyperammonemia and neuroinflammation have been considered to play synergistic roles in inducing neurological manifestations of HE [25]. Nevertheless, previous validation studies have failed to show a consistent relationship between high blood ammonia concentrations and MHE [14,26,27]. On the contrary, elevated levels of serum cytokines have been repeatedly reported in patients with chronic liver disease [28,29]. Previous literatures have revealed that IL-6 and IL-1β showed significant correlations with PHES score and correlate with health-related quality of life irrespective of MHE [30] Serum IL-6 and IL-18 levels are reported to be correlated with the degree of encephalopathy [6,31]. We also identified that higher serum IL-6 levels can be independently predictive of MHE, which may support that IL-6 had a more robust association with cognitive impairment in liver cirrhosis [32]. Furthermore, IL-6 is linked with cognitive decline among the older participants, independent of other vascular risk factors such as C-reactive protein (CRP).[33] Higher IL-6 level in midlife was also associated with cognitive decline. These evidences suggested that IL-6 might be a more appropriate marker for cognition impairment, such as the MHE in the current study [34] Although literature has shown an increased serum level of TNF-α in cirrhotic patients with HE [35–37], we did not detect such a phenomenon in the current study. We suggest that the severity of cirrhosis may have accounted for this discrepancy, because most of our cirrhotic patients were of Child-Pugh grade A status. And previous report has shown that serum TNF-α levels correlate with the severity of cirrhosis [38,39]. In addition, one study even demonstrated that TNF-α could contribute to OHE, but not MHE [40]. Thus, IL-6 is independently predictive of MHE occurrence, and may have the potential to be the objective biomarker of MHE. [6,41].

After test and re-test, we found that the PHES is a reliable tool to detect neuropsychiatric dysfunction. Correlation coefficients exceeded 0.80 for the DST, NCT-A, NCT-B, and LTT tests, but not for the SDT test (0.73). However, we also observed a learning effect between the 2 tests, which agrees with previous findings from Italy and Mexico. Therefore, we should be cautious when using PHES tests repeatedly in the same patients.

Limitations do exist in this study. First, we did not examine the relationship between serum ammonia level and MHE. Although ammonia is considered to play a role in the pathogenesis of HE/MHE [20], no consistent correlation can be clearly identified between the blood ammonia level and the presenting symptoms of HE [1,42]. Thus, its use in diagnosing HE is not recommended [43]. Nevertheless, a recent study has identified a positive correlation between serum IL-6 levels and plasma ammonia levels in cirrhotic patients with OHE and suggested that IL-6 might be involved in the mechanism by which ammonia contributes to the pathogenesis of HE.[44] Secondly, in the current study cohort, only one patient had been diagnosed as cirrhosis by liver biopsy. The others were clinically diagnosed by blood tests (low platelet count), endoscopic finding (presence of varices), and abdominal imaging study (splenomegaly with signs of liver cirrhosis in ultrasonography and/or fibroscan). Traditionally, pathologic examination of percutaneous biopsy specimens is considered to be the golden standard for diagnosis of chronic liver disease including cirrhosis. But, this procedure is invasive and not indicated for patients with bleeding tendency and ascites. Furthermore, the ‘non-invasive’ diagnosis of hepatic fibrosis in patients with chronic liver disease has progressed significantly recently and have shown high diagnostic accuracy for significant fibrosis and liver cirrhosis. [45] Thus, the need for liver biopsy has been challenged in many clinical situations where patients have chronic hepatitis B or C. It is suggested that the need for liver biopsy should be performed when there is sufficient uncertainty about diagnosis, severity of disease, prognosis, and treatment decisions.[46] Therefore, clinical diagnosis instead of liver biopsy was mainly used for cirrhosis in all recent studies in the PHES validation for MHE. However, we agreed that this approach may underestimate the diagnosis of liver cirrhosis resulting in overestimation of MHE prevalence. Finally, all of our cirrhotic patients were Taiwanese outpatients who mainly had mild to moderate disease severity. This hinders the generalizability of our findings to patients with acute liver disease, patients of other nationalities, or patients hospitalized with advanced cirrhosis.

In summary, the PHES can be a simple and useful tool for detecting MHE among cirrhotic patients in Taiwan. Around one third of cirrhotic patients from an outpatient clinic were identified to have MHE based on a PHES score < -4. High serum IL-6 levels are predictive of the presence of MHE among cirrhotic patients.
